# Changes in period and cohort effects on haematological cancer mortality in Spain, 1952-2006

**DOI:** 10.1186/1471-2407-14-250

**Published:** 2014-04-10

**Authors:** Roberto Pastor-Barriuso, Gonzalo López-Abente

**Affiliations:** 1 National Center for Epidemiology, Carlos III Institute of Health, Monforte de Lemos 5, 28029, Madrid, Spain; 2 Consortium for Biomedical Research in Epidemiology and Public Health (CIBERESP), Melchor Fernández Almagro 3-5, 28029, Madrid, Spain

**Keywords:** Haematological cancer mortality, Period and cohort effects, Change-points, Diagnosis and certification improvements

## Abstract

**Background:**

In contrast to other haematological cancers, mortality from non-Hodgkin’s lymphoma and multiple myeloma increased dramatically during the second half of the 20th century in most developed countries. This widespread upward trend remains controversial, as it may be attributable either to progressive improvements in diagnosis and certification or to increasing exposures to little-known but relevant risk factors.

**Methods:**

To assess the relative contribution of these factors, we analysed the independent effects of age, death period, and birth cohort on haematological cancer mortality rates in Spain across the period 1952-2006. Weighted joinpoint regression analyses were performed to detect and estimate changes in period and cohort curvatures.

**Results:**

Although mortality rates were consistently higher among men, trends across periods and cohorts were virtually identical in both sexes. There was an early period trend reversal in the 1960s for Hodgkin’s disease and leukaemia, which was delayed to the 1980s for multiple myeloma and the 1990s for non-Hodgkin’s lymphoma. Birth cohort patterns showed a first downturn for generations born in the 1900s and 1910s for all haematological cancers, and a second trend reversal for more recent cohorts born in the 1950s and 1960s for non-Hodgkin’s lymphoma and leukaemia.

**Conclusions:**

The sustained decline in Hodgkin’s disease mortality and the levelling off in leukaemia seem to be driven by an early period effect linked to improvements in disease treatment, whereas the steep upward trends in non-Hodgkin’s lymphoma and multiple myeloma mortality in Spain are more likely explained by a cohort effect linked to better diagnosis and death certification in the elderly. The consistent male excess mortality across all calendar periods and age groups points to the importance of possible sex-related genetic markers of susceptibility in haematological cancers.

## Background

Haematological malignancies constituted 6.9% of all incident cancer cases and 7.2% of all cancer deaths in Spain in 2012 [1]. Morbidity and mortality from haematological cancers in Spain are fairly similar to the European average [2], with age-adjusted incidence and mortality rates of 2.4 cases and 0.3 deaths per 100,000 person-years for Hodgkin’s disease, 10.0 cases and 3.3 deaths for non-Hodgkin’s lymphoma (NHL), 3.5 cases and 2.2 deaths for multiple myeloma, and 8.3 cases and 4.4 deaths for leukaemia.

Since the mid-1970s, mortality from Hodgkin’s disease and leukaemia declined steadily in most Western European countries [3,4], a downward trend that was mainly attributable to substantial improvements in disease treatment. In contrast, in the second half of the 20th century, there was a dramatic increase in incidence and mortality from NHL and multiple myeloma in many European countries [3,5-7] and North America [8,9], which has only begun to level off in recent years [10-13]. The reasons for this widespread upward trend in developed countries are not fully understood, since it may be largely artefactual due to the progressive implementation of modern diagnostic imaging techniques over recent decades, or it may partly reflect a real increase in underlying incidence linked to changes in environmental exposures to lymphomagenic agents and other unknown risk factors. In addition, lymphoid tumours have shown several problems of case ascertainment and death certification from the beginning of cancer registry activities, which further complicated the interpretation of time trends in these haematological cancers [14].

Age-period-cohort models allow to disentangle the independent effects of age, death period, and birth cohort on long-term time trends in cancer mortality, after controlling for the potential confounding effects of the remaining factors [15]. In particular, the identification of significant trend changes in period and cohort effects may help explain some features of the observed time trends in haematological cancer mortality and assess the relative contribution of both long-term exposure to risk factors and recent improvements in diagnosis and treatment. To this end, the present study sought to analyse changes in period and cohort effects on haematological cancer mortality in Spain across the period 1952-2006.

## Methods

### Data source

The number of deaths from Hodgkin’s disease, NHL, multiple myeloma, and leukaemia in Spain, broken down by single calendar year, sex, and 5-year age group (0-4, 5-9, …, 80-84, and ≥ 85 years), was obtained from the Spanish National Institute of Statistics for the period 1952-2006 [16]. The study period was restricted to 1962-2006 for multiple myeloma, due to the reduced number of deaths from this cause registered in Spain before 1962. Deaths from these haematological cancers corresponded to different codes from successive revisions of the International Classification of Diseases (ICD) over the study period (Additional file 1: Table S1). Haematological cancer death certification in Spain has been shown to be accurate during the 1980s and 1990s, with detection and confirmation rates of 86% and 80% for lymphomas, 96% and 94% for multiple myeloma, and 93% and 93% for leukaemia, as compared with clinical and pathological reports [17]. Mid-year population estimates for Spain were also obtained from the Spanish National Institute of Statistics from 1952 to 2006 [16].

### Statistical analysis

Age-adjusted mortality rates of haematological cancers were calculated for each sex and 5-year calendar period (1952-1956 to 2002-2006) using the direct method with the European standard population. For each haematological cancer, age-adjusted male-to-female mortality rate ratios and their 95% confidence intervals (CIs) were computed for each 5-year period by assuming a Poisson distribution for the observed number of deaths.

To assess the effect of age, death period, and birth cohort on time trends in haematological cancer mortality in Spain, log-linear Poisson models [18] were fitted to data aggregated by 5-year age group and calendar period. The open-ended group of persons aged 85 years or older was excluded from these age-period-cohort analyses, as well as those younger age groups having fewer than five deaths in any 5-year calendar period. To overcome the problem of non-identifiability of model parameters arising from the exact linear dependence among age, period, and cohort, we adopted the approach proposed by Holford [15,19] and considered estimable functions of parameters, such as the curvatures in each effect and the sum of period and cohort linear slopes, also known as the net drift. Although other solutions to the non-identifiability problem have been proposed [15], the net drift and curvatures are uniquely determined by the data and hence remain invariant irrespective of the particular approach used. More specifically, the number of deaths from each haematological cancer and sex *d*_*ap*_ registered at age *a* in period *p* was assumed to follow a Poisson distribution with mean *λ*_*ap*_*n*_*ap*_ and free dispersion parameter *ϕ*, where *λ*_*ap*_ is the underlying mortality rate and *n*_*ap*_ is the number of person-years at risk. The general form of the log-linear age-period-cohort model for rates *λ*_*ap*_ at age *a* in period *p* for persons in birth cohort *c* = *p* – *a* was

$$\log\left(\lambda_{\mathrm{ap}}\right)=\mu +\alpha_{a}+\beta_{p}+\gamma_{c},$$

where the intercept *μ* is the average log-rate over all age groups and calendar periods, and the parameters *α*_*a*_, *β*_*p*_, and *γ*_*c*_ represent the deviations from this overall log-rate for age *a*, period *p*, and cohort *c*, whose respective averages (weighted by the corresponding marginal number of person-years) are constrained to be 0. To address the linear relationship between age, period, and cohort, each temporal effect was further partitioned into linear and curvature components [19,20]. The age effect was decomposed as $\alpha_{a}=\alpha_{l}\left(a-{\bar{a}}_{w}\right)+{\tilde{\alpha }}_{a}$, where *α*_*l*_ is the age slope, ${\bar{a}}_{w}$ is the average age index (weighted by the marginal number of person-years by age group), and ${\tilde{\alpha }}_{a}$ is the age curvature with the linear trend removed. Applying similar partitions to the period and cohort effects, the model was reparameterized as

$$\begin{array}{l} \log\left(\lambda_{\mathrm{ap}}\right)=\mu +\{\alpha_{l}(a-{\bar{a}}_{w})+{\tilde{\alpha }}_{a}\}\\ \;\;+\{\beta_{l}(p-{\bar{p}}_{w})+{\tilde{\beta }}_{p}\}+\{\gamma_{l}(c-{\bar{c}}_{w})+{\tilde{\gamma }}_{c}\}\\ \;=\mu +\left(\alpha_{l}-\gamma_{l}\right)(a-{\bar{a}}_{w})+(\beta_{l}+\gamma_{l})(p-{\bar{p}}_{w})+{\tilde{\alpha }}_{a}\\ \;\;+{\tilde{\beta }}_{p}+{\tilde{\gamma }}_{c}, \end{array}$$

since $c-{\bar{c}}_{w}=\left(p-{\bar{p}}_{w}\right)-\left(a-{\bar{a}}_{w}\right)$ due to the linear dependence among the three factors. All parameters of this model are identifiable and hence have unique maximum likelihood estimates [19]. This paper focused on the cross-sectional age effect for an average period [20], which was obtained from the linear function $\mu +\left(\alpha_{l}-\gamma_{l}\right)\left(a-{\bar{a}}_{w}\right)+{\tilde{\alpha }}_{a}$, the net drift or sum of period and cohort linear slopes *β*_*l*_ + *γ*_*l*_, and the period and cohort curvatures ${\tilde{\beta }}_{p}$ and ${\tilde{\gamma }}_{c}$.

To detect and estimate changes in the period and cohort effects of the three-factor model, separate joinpoint regression analyses of the estimated period and cohort curvatures ${\tilde{\beta }}_{p}$ and ${\tilde{\gamma }}_{c}$ were performed with weights inversely proportional to their estimated variances *v*_*p*_ and *v*_*c*_ from the above age-period-cohort model [21]. These joinpoint models provided the number of significant change-points across periods and cohorts by using permutation tests, the estimate and 95% CI for the location of each change-point, and the estimate and 95% CI for the difference in annual percent changes (APCs) before and after each change-point. Specifically, the joinpoint regression model for the period curvature ${\tilde{\beta }}_{p}$ with a single significant change-point *τ* was

$${\tilde{\beta }}_{p}=\beta_{0}+\beta_{1}p+\beta_{2}{\left(p-\tau \right)}_{+}+\epsilon ,$$

where (*p* – *τ*)_+_ = *p* – *τ* if the period *p* is above the change-point *τ* and 0 if *p* is below *τ*, and *ϵ* is the error with mean 0 and heterogeneous variance *v*_*p*_[21]. To interpret joinpoint regression coefficients, we added back the period linear trend to the period curvature from the above joinpoint model and obtained the two-segmented period effect

$$\begin{array}{l} \beta_{p}=\beta_{l}\left(p-{\bar{p}}_{w}\right)+{\tilde{\beta }}_{p}\\ \;\;=\beta_{0}^{*}+\left(\beta_{l}+\beta_{1}\right)p+\beta_{2}{\left(p-\tau \right)}_{+}+\epsilon , \end{array}$$

where the intercept $\beta_{0}^{*}=\beta_{0}-\beta_{l}{\bar{p}}_{w}$. The parameters *β*_*l*_ + *β*_1_ and *β*_*l*_ + *β*_1_ + *β*_2_ represented the period slopes below and above the change-point *τ*, respectively, which were not estimable since the overall period slope *β*_*l*_ cannot be uniquely determined [19]. However, the change in period slopes at the change-point can be estimated directly as the coefficient *β*_2_ from joinpoint regression analysis of period curvatures. Similar arguments can be applied to cohort effects, as well as to the presence of multiple change-points.

## Results

### Time trends

**Figure 1:**
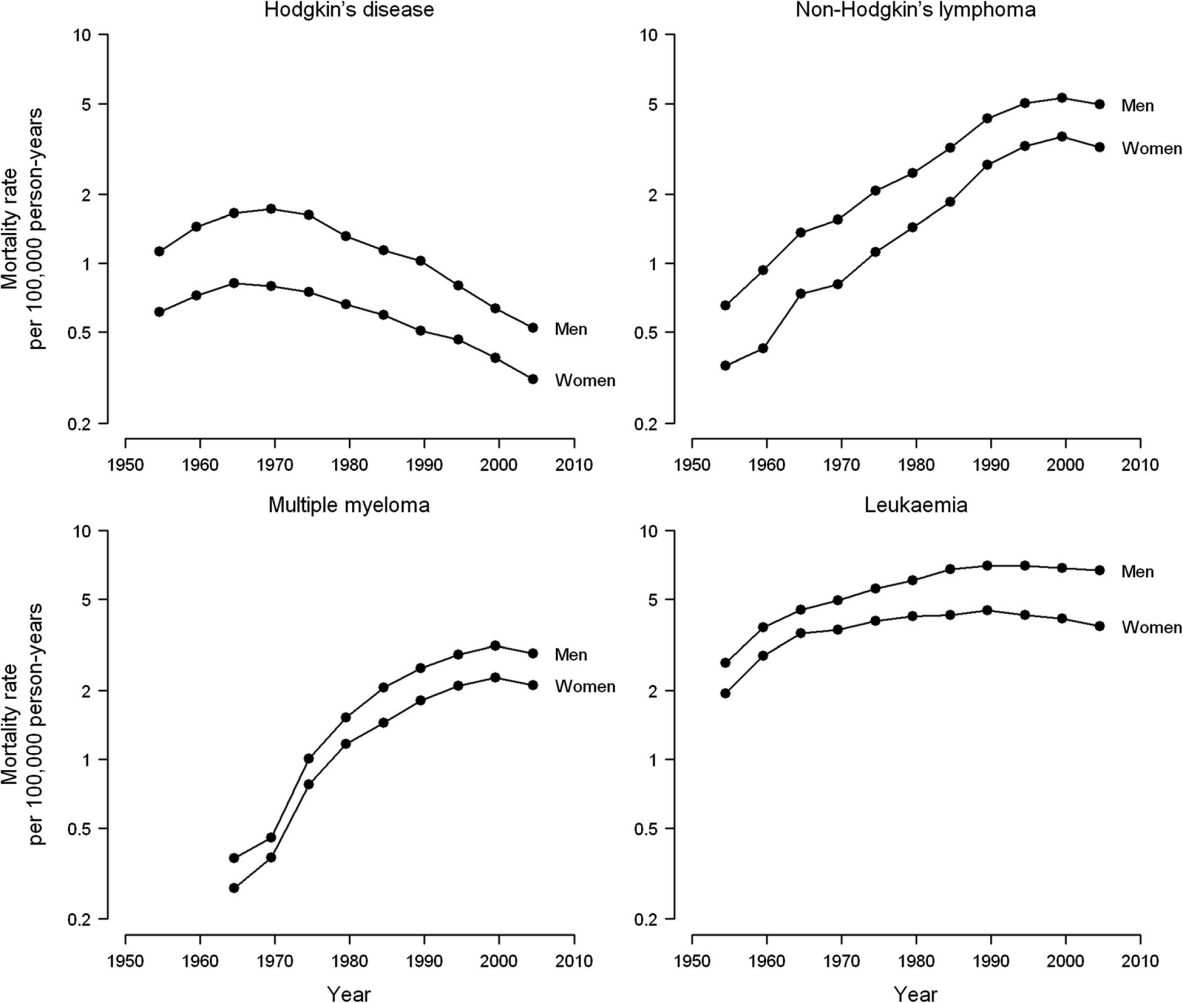
Age-adjusted mortality rates of haematological cancers (per 100,000 European standard population) by sex, Spain 1952-2006.

Age-adjusted mortality rates of haematological cancers were consistently higher among men than among women over all 5-year periods, with male-to-female rate ratios ranging from 1.65 to 2.17 for Hodgkin’s disease, 1.48 to 2.20 for NHL, 1.22 to 1.43 for multiple myeloma, and 1.27 to 1.75 for leukaemia (Table 1). Time trends were similar in both sexes, but differed substantially for each haematological cancer (Figure 1). Hodgkin’s disease mortality declined sharply after a short, initial upward trend in the 1950s and 1960s (APCs in age-adjusted rates over the entire study period of -2.3% in men and -1.8% in women). However, mortality rates of NHL and multiple myeloma rose steeply throughout the 20th century and attenuated only in recent years (overall APCs of 2.3% in men and 3.0% in women for NHL, and 3.6% in both men and women for multiple myeloma). The initial upward trend in leukaemia mortality gradually levelled off and tended to decline as from the 1990s onwards (overall APCs of -0.1% in men and -0.3% in women).

**Table 1:** Age-adjusted mortality rates of haematological cancers in Spain (per 100,000 European standard population) by sex and 5-year calendar period

	**1952–1956**		**1957–1961**		**1962–1966**		**1967–1971**		**1972–1976**		**1977–1981**		**1982–1986**		**1987–1991**		**1992–1996**		**1997–2001**		**2002–2006**	
**Haematological cancer**	**No. of deaths**	**Rate**	**No. of deaths**	**Rate**	**No. of deaths**	**Rate**	**No. of deaths**	**Rate**	**No. of deaths**	**Rate**	**No. of deaths**	**Rate**	**No. of deaths**	**Rate**	**No. of deaths**	**Rate**	**No. of deaths**	**Rate**	**No. of deaths**	**Rate**	**No. of deaths**	**Rate**
**Hodgkin’s disease**																						
Men	659	1.13	894	1.45	1,091	1.65	1,214	1.73	1,201	1.63	1,052	1.32	982	1.14	951	1.03	794	0.80	698	0.63	647	0.52
Women	406	0.61	510	0.72	624	0.82	642	0.80	633	0.75	625	0.66	614	0.60	583	0.51	592	0.46	538	0.39	496	0.31
Male-to-female ratio^1^ (95% CI)		1.84 (1.62–2.09)		2.01 (1.80–2.24)		2.01 (1.82–2.22)		2.17 (1.97–2.39)		2.17 (1.97–2.40)		1.98 (1.80–2.19)		1.92 (1.73–2.12)		2.03 (1.82–2.25)		1.73 (1.55–1.93)		1.65 (1.46–1.85)		1.67 (1.48–1.89)
**Non-Hodgkin’s lymphoma**																						
Men	360	0.65	554	0.93	875	1.36	1,098	1.55	1,540	2.07	1,982	2.47	2,736	3.19	3,992	4.30	5,046	5.00	5,957	5.29	6,272	4.97
Women	232	0.36	295	0.42	555	0.74	660	0.81	972	1.12	1,369	1.43	2,022	1.85	3,226	2.70	4,394	3.25	5,457	3.58	5,652	3.21
Male-to-female ratio^1^ (95% CI)		1.82 (1.54–2.16)		2.20 (1.90–2.54)		1.84 (1.65–2.05)		1.91 (1.73–2.11)		1.85 (1.71–2.01)		1.72 (1.61–1.85)		1.72 (1.62–1.82)		1.59 (1.52–1.67)		1.54 (1.47–1.60)		1.48 (1.42–1.54)		1.55 (1.49–1.61)
**Multiple myeloma**																						
Men					215	0.37	290	0.46	679	1.01	1,132	1.52	1,731	2.06	2,351	2.51	2,949	2.87	3,660	3.13	3,881	2.90
Women					197	0.27	294	0.37	683	0.78	1,145	1.16	1,642	1.44	2,311	1.81	2,984	2.09	3,717	2.27	3,920	2.11
Male-to-female ratio^1^ (95% CI)						1.35 (1.11–1.64)		1.22 (1.04–1.44)		1.30 (1.16–1.44)		1.30 (1.20–1.42)		1.43 (1.33–1.53)		1.39 (1.31–1.47)		1.37 (1.30–1.45)		1.38 (1.31–1.44)		1.38 (1.31–1.44)
**Leukaemia**																						
Men	1,592	2.63	2,435	3.77	3,128	4.50	3,648	4.93	4,224	5.56	4,927	6.06	5,881	6.75	6,562	7.03	7,132	7.03	7,748	6.86	8,547	6.67
Women	1,338	1.94	2,097	2.82	2,778	3.55	3,073	3.67	3,576	4.01	4,109	4.21	4,630	4.27	5,316	4.45	5,674	4.27	6,166	4.12	6,609	3.81
Male-to-female ratio^1^ (95% CI)		1.36 (1.26–1.46)		1.33 (1.26–1.42)		1.27 (1.20–1.33)		1.34 (1.28–1.41)		1.39 (1.32–1.45)		1.44 (1.38–1.50)		1.58 (1.52–1.64)		1.58 (1.52–1.64)		1.65 (1.59–1.71)		1.67 (1.61–1.73)		1.75 (1.69–1.81)

^1^Age-adjusted mortality rate ratio of males to females and 95% confidence interval (CI).

### Effect of age, death period, and birth cohort

**Figure 2:**
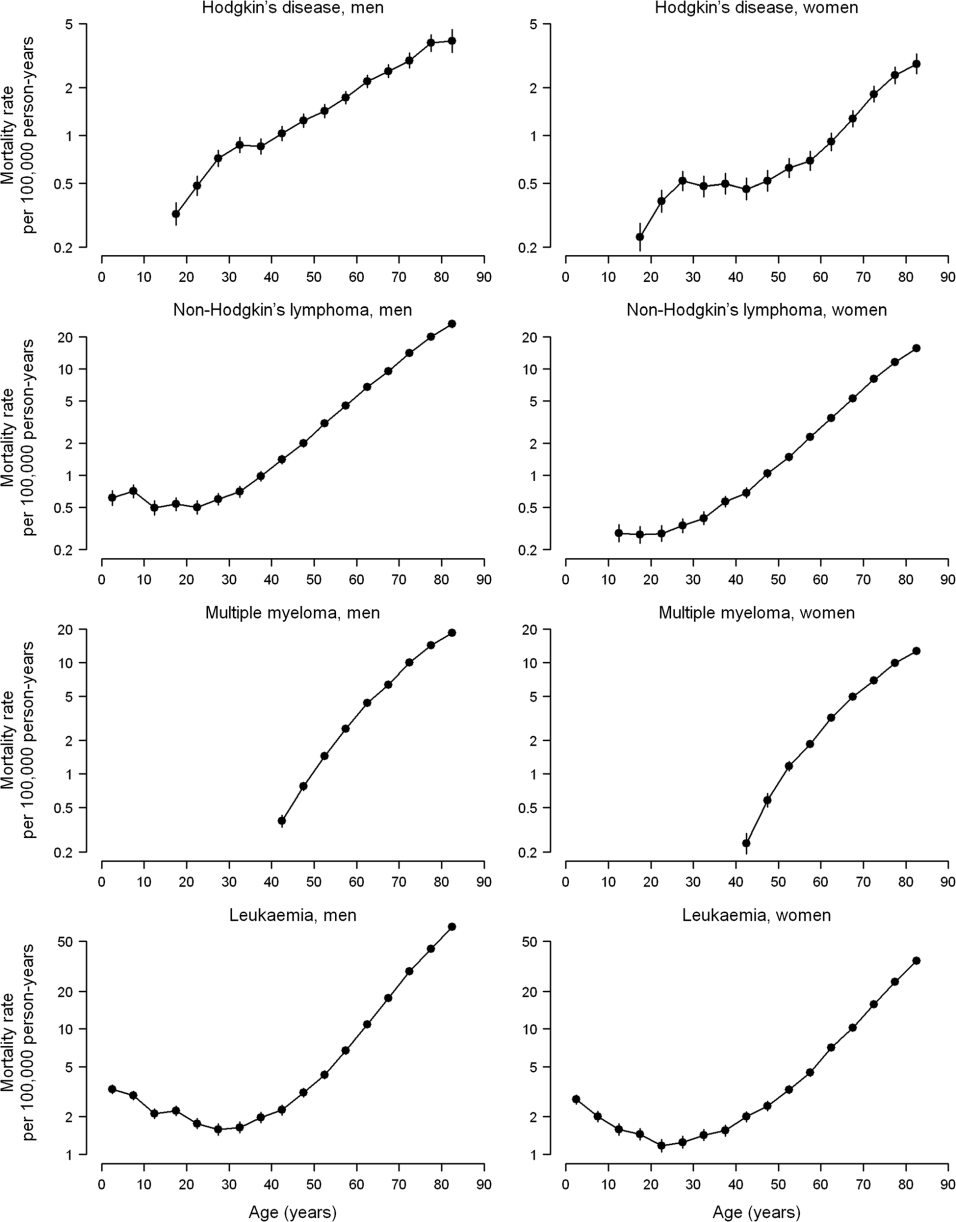
**Cross-sectional age effect on haematological cancer mortality rates by sex, Spain 1952-2006.** The cross-sectional age effects for an average period and their 95% confidence intervals (vertical bars) were obtained from the three-factor model with period-allocated drift and period and cohort curvatures.

The cross-sectional age effects on haematological cancer mortality are displayed in Figure 2. The increasing age trend in Hodgkin’s disease mortality rates showed a deflection at age 30, which was more marked in women than in men. NHL and leukaemia mortality increased log-linearly with age among middle-aged and older adults, with a flat relation at younger ages for NHL and a J-shaped trend for leukaemia. Mortality from multiple myeloma also increased with age but showed a gradual deceleration at older ages.

**Figure 3:**
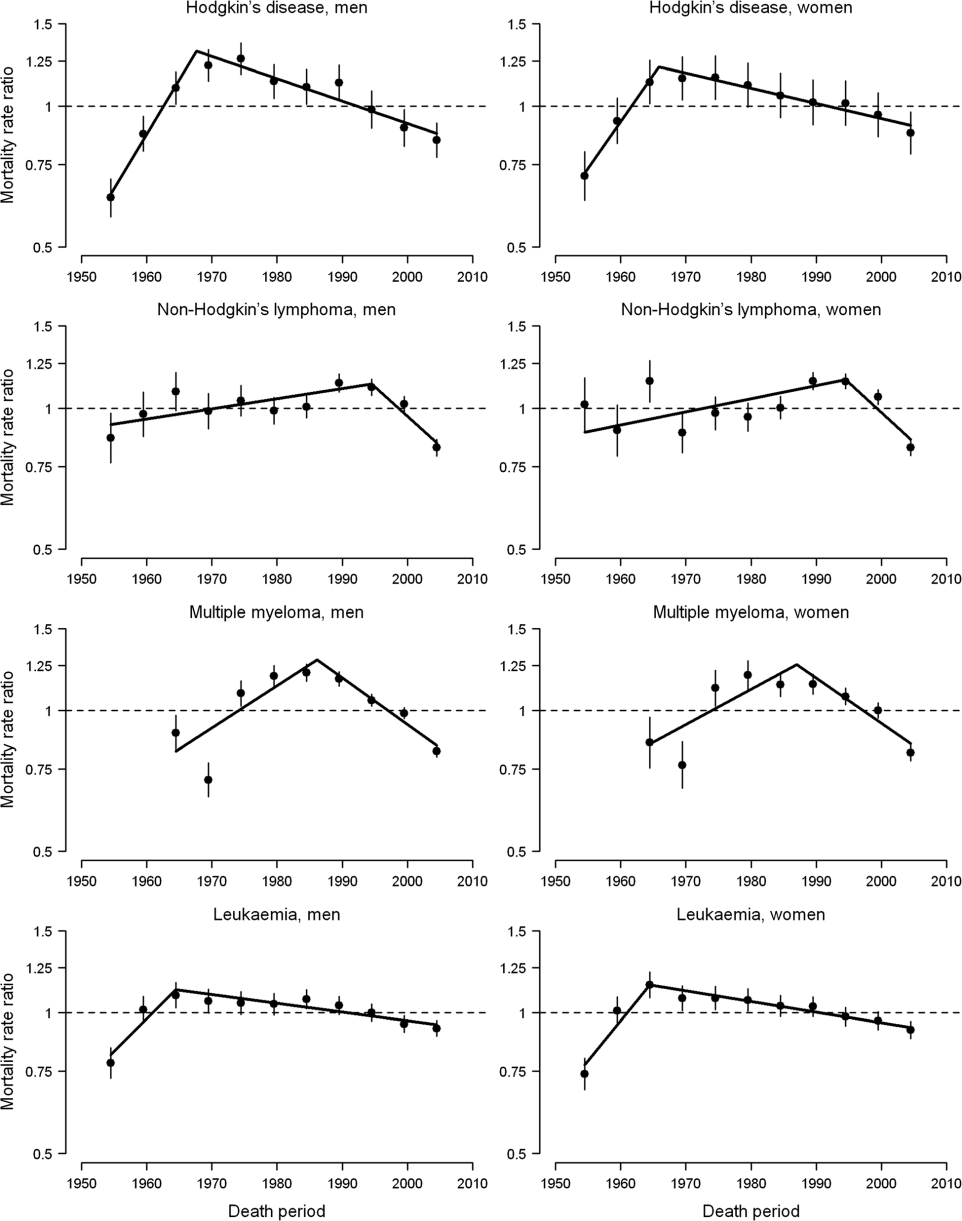
**Curvature in period effect on haematological cancer mortality rates by sex, Spain 1952-2006.** The period curvatures and their 95% confidence intervals (vertical bars) were obtained from the three-factor model with the period linear slope removed. Significant trend changes (connected solid segments) were obtained from weighted joinpoint regression analyses of period curvatures.

The curvatures in period effects, as well as the significant period trend changes obtained from joinpoint regression analyses, are shown in Figure 3 and Table 2. Period curvatures for each haematological cancer were virtually identical in both sexes. Hodgkin’s disease mortality experienced a sharp period trend reversal in the 1960s (APC decreases of -6.3% in 1967 for men and -5.2% in 1965 for women), an early pattern that was also observed to a lesser extent for leukaemia (APC decreases of -3.6% and -4.4% in 1964 for men and women, respectively). This favourable evolution in period trends was delayed to the 1980s for multiple myeloma (APC decreases of -4.3% in 1986 for men and -3.9% in 1987 for women) and to the 1990s for NHL (APC decreases of -3.3% and -3.5% in 1994 for men and women, respectively).

**Table 2:** Changes in period and cohort effects on haematological cancer mortality rates by sex, Spain 1952-2006

	**Net drift**^**1**^	**Change in period effect**^**2**^		**Change in cohort effect**^**3**^			
**Haematological cancer**		**Year of death**	**Difference in**	**Birth year**	**Difference in**	**Birth year**	**Difference in**
	**(95% CI)**	**(95% CI)**	**APC (95% CI)**	**(95% CI)**	**APC (95% CI)**	**(95% CI)**	**APC (95% CI)**
**Hodgkin’s disease**							
Men	-2.3 (-2.6 to -2.1)	1967.7 (1965.5–1972.5)	-6.3 (-7.5 to -5.0)	1914.1 (1904.0–1919.0)	-2.3 (-2.9 to -1.7)	—	—
Women	-1.8 (-2.0 to -1.5)	1965.9 (1965.0–1968.0)	-5.2 (-6.0 to -4.4)	1915.1 (1909.5–1919.0)	-3.5 (-4.8 to -2.2)	1932.0 (1927.0–1976.5)	1.5 (0.2 to 2.9)
**Non-Hodgkin’s lymphoma**							
Men	2.3 (2.0 to 2.5)	1994.5 (1990.0–NA)	-3.3 (-5.1 to -1.5)	1919.9 (1915.0–1924.5)	-3.6 (-4.1 to -3.1)	1968.7 (1962.0–1975.0)	-4.9 (-6.2 to -3.6)
Women	3.0 (2.7 to 3.3)	1994.5 (1989.0–NA)	-3.5 (-6.0 to -1.0)	1918.7 (1917.0–1920.5)	-4.3 (-4.6 to -3.9)	1954.1 (1945.5–1962.0)	-2.3 (-2.9 to -1.6)
**Multiple myeloma**							
Men	3.6 (3.4 to 3.9)	1986.1 (1975.5–1994.0)	-4.3 (-6.0 to -2.6)	1909.1 (1905.0–1911.5)	-6.1 (-6.9 to -5.2)	1928.9 (1923.5–1936.5)	-1.8 (-2.3 to -1.3)
Women	3.6 (3.2 to 3.9)	1987.0 (1975.0–1994.0)	-3.9 (-5.7 to -2.0)	1905.4 (1902.5–1910.0)	-6.1 (-7.8 to -4.5)	1924.7 (1922.0–1930.0)	-2.9 (-3.5 to -2.3)
**Leukaemia**							
Men	-0.1 (-0.2 to 0.1)	1964.5 (NA–1969.5)	-3.6 (-5.8 to -1.3)	1913.4 (1907.5–1916.5)	-4.3 (-4.9 to -3.7)	1964.6 (1939.5–1973.0)	-2.5 (-3.3 to -1.7)
Women	-0.3 (-0.5 to -0.2)	1964.5 (NA–1967.0)	-4.4 (-5.7 to -3.0)	1910.1 (1907.5–1915.5)	-5.1 (-5.8 to -4.4)	1966.2 (1953.5–1972.5)	-2.8 (-3.6 to -2.0)

^1^Overall annual percent change in age-adjusted mortality rates and 95% confidence interval (CI) obtained from the sum of period and cohort linear slopes from the three-factor model. ^2^Difference in annual percent changes (APCs) before and after the estimated year of death with significant trend change as obtained from the weighted joinpoint regression analysis of period curvatures from the three-factor model. ^3^Differences in annual percent changes (APCs) before and after the estimated birth years with significant trend changes as obtained from the weighted joinpoint regression analysis of cohort curvatures from the three-factor model.

**Figure 4:**
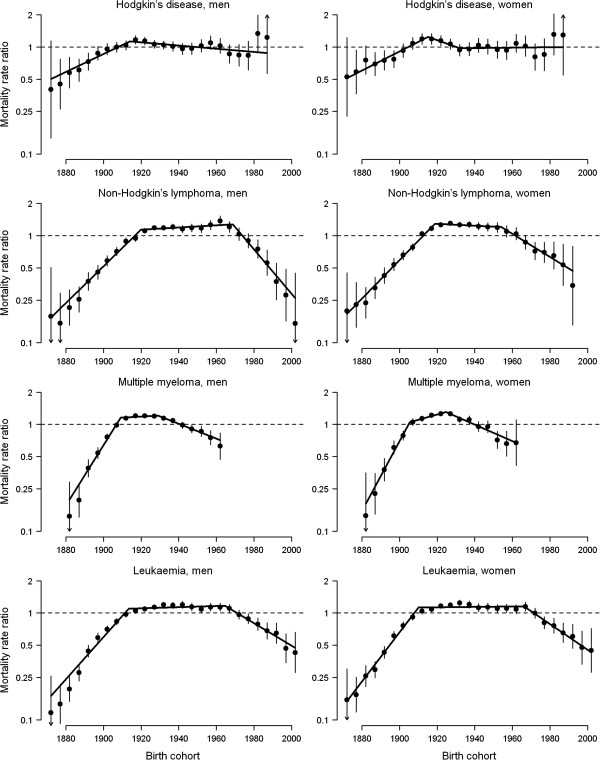
Curvature in cohort effect on haematological cancer mortality rates by sex, Spain 1952-2006 **Curvature in cohort effect on haematological cancer mortality rates by sex, Spain 1952-2006.** The cohort curvatures and their 95% confidence intervals (vertical bars) were obtained from the three-factor model with the cohort linear slope removed. Significant trend changes (connected solid segments) were obtained from weighted joinpoint regression analyses of cohort curvatures.

The curvatures and significant trend changes in cohort effects are shown in Figure 4 and Table 2. Birth cohort patterns were similar for both sexes and exhibited a first downturn in mortality trends for generations born in the first two decades of the 1900s, which was more marked for multiple myeloma (APC decrease of -6.1% in both sexes) than for the other haematological cancers (APC decreases of -4.3% to -2.3% in men and -5.1% to -3.5% in women). A second, more recent trend reversal was also detected for cohorts born in the 1950s and 1960s for NHL (APC decreases of -4.9% for men born in 1968 and -2.3% for women born in 1954) and leukaemia (APC decreases of -2.5% for men born in 1964 and -2.8% for women born in 1966).

## Discussion

### Main findings

Our results show that, although mortality rates from haematological malignancies were consistently higher among men than among women throughout the extensive period studied, death period and birth cohort patterns were virtually identical in both sexes. In the case of period effects, the change-point at which mortality attenuated was located in different calendar years, depending on the cause of death. Hodgkin’s disease and leukaemia were the first tumours displaying this attenuation in the 1960s, followed by multiple myeloma in the 1980s and NHL in the 1990s. The early period trend reversal for Hodgkin’s disease and leukaemia was essentially attributable to substantial improvements in the treatment of these haematological tumours, as has already been documented in other European countries [3,4].

With respect to cohort effects, two findings should be highlighted, namely, the homogeneous upward trends for generations born before the 1920s for all four haematological tumours and both sexes, and the similar inverted U-shaped trends for NHL and leukaemia across all birth cohorts. The widespread rises in earlier cohorts suggest a phenomenon of progressive improvement and subsequent saturation in the diagnosis and certification of haematological tumours, particularly in the elderly. It is also noteworthy that the decline in NHL mortality for more recent generations occurred a decade later in men than in women, since men born around 1960 were most affected by the human immunodeficiency virus epidemic derived from injection drug use.

### Joinpoint analyses of period and cohort curvatures

Joinpoint analyses of age-adjusted rates are commonly used to identify significant trend changes over a given period. However, with this approach, we are unable to disentangle whether the observed trend changes across periods are due to a unique period effect or to an indirect effect induced by birth cohorts [15]. We therefore conducted weighted joinpoint analyses of the estimated period and cohort curvatures from the three-factor model, rather than relying directly on age-adjusted rates. Although curvatures are hard to interpret in isolation from the overall linear slopes [22], the locations of change-points and the associated changes in period and cohort slopes remain estimable from curvature components.

### Non-Hodgkin’s lymphoma and multiple myeloma mortality trends

The steady rise in mortality from haematological cancers until the generations born in the 1920s and the subsequent levelling off would suggest that diagnosis and, by extension, certification of these diseases increase as health care improves and develops. While there may feasibly be a strong chance of diagnostic enhancements [12,13,23] and universal access to health care in Spain being incorporated into the dramatic increases in NHL and multiple myeloma rates, it has been argued that diagnostic improvements cannot fully explain the widespread rise in NHL incidence until the end of the 1990s in other countries, with the most likely explanation being an increase in exposure to risk factors [24]. If the uniform escalation in NHL incidence and mortality occurred due to greater exposure to risk factors of different nature, then such factors should not only be strongly associated with NHL risk, but should also be widely distributed across all adult age groups, both sexes, and most developed countries. Severe immunosuppression and certain infectious agents are the only well-established, strong risk factors for NHL [24], but these conditions are too infrequent in the general population to account for a substantial part of the widespread rise in NHL rates. Weaker and less consistent associations with NHL risk have been reported for occupational and environmental exposures to chemicals, pesticides, and persistent organic pollutants, as well as for several dietary and lifestyle factors, including high intake of fats and meat, overweight, and smoking for specific NHL subtypes [25]. While exposure to chemicals and pesticides has mainly been occupational and hence unevenly distributed between sexes and age groups, the whole population in developed countries was uniformly and increasingly exposed to persistent organic pollutants and unhealthy lifestyle habits during the second half of the 20th century. The slight but sustained upward period effects on NHL mortality observed in Spanish men and women up to the middle 1990s could be attributable to increased exposure to these potential risk factors, as has been suggested for NHL incidence in other European countries [26]. Nevertheless, our results show a more prominent role of birth cohort, rather than death period, in defining the dramatic upward trends in NHL and multiple myeloma mortality in Spain. The sharp rises for earlier generations of both sexes suggest that progressive improvements in case ascertainment and certification in the elderly have probably played a major role in the dramatic increase in mortality from haematological tumours. In line with these countrywide mortality findings, previous studies on NHL incidence trends in several Spanish regions have also reported a recent attenuation of the long-term epidemic increase in NHL [6,13], which was mainly attributable to the progressive introduction and subsequent full implementation of modern diagnostic procedures.

### Hodgkin’s disease and leukaemia mortality trends

As in most Western European countries [3,12,27], mortality from Hodgkin’s disease has declined markedly in Spain during the last decades, since this neoplasm is highly amenable to treatment [28]. The continued downward period effects after the middle 1960s, together with the relatively flat cohort effects, indicate that advancements in the integrated treatment of Hodgkin’s disease had a favourable impact in all age groups. With regard to leukaemia, our results are consistent with previous studies in showing that there has been considerable progress in its treatment among children and young adults [4,29], as indicated by the downward trends across periods and recent generations born after the middle 1960s. However, the favourable effect of leukaemia treatment in young patients was partially offset by the increase in mortality linked to better diagnosis and certification in earlier cohorts born before the 1920s [3], thus resulting in an overall levelling off in leukaemia mortality rates from 1990 onwards.

### Male excess mortality from haematological cancers

It is very striking that, despite the markedly different time trends in mortality from each haematological cancer, male-to-female mortality rate ratios remained fairly constant throughout the entire study period for all haematological tumours. The male excess mortality persisted not only across successive calendar periods, but also across all age groups, including children younger than 15 years (see age- and period-specific mortality rates of NHL and leukaemia in Additional file 2: Video S1 and Additional file 3: Video S2). The same consistent predominance among men has been reported for NHL incidence in Spain [13], as well as for the incidence of all four haematological tumours in different countries around the world [30]. Lifestyle and environmental factors known to be unevenly distributed between sexes, such as smoking, alcohol drinking, and occupational carcinogens, can hardly account for these enigmatic sex disparities in haematological cancer incidence and mortality [30], giving way to endogenous sex-specific biological or genetic factors as a more plausible explanation. The existence of X-chromosome-linked suppressor factors of haematological tumours has recently been suggested, something that would help explain the systematic lower mortality observed in women. In particular, the loss of the *PHF6* tumour suppressor gene, linked to the X chromosome, has been identified as possibly being involved in the genesis of T-cell acute lymphoblastic leukaemia [31] and adult acute myeloid leukaemia [32], though neither paper reports the frequency of this disorder in the general population. While it is uncertain whether these findings on very specific types of lymphomas and leukaemia could be generalised to other lymphoid neoplasms and leukaemias, they have already been observed for some solid tumours [33].

### Limitations of the study

Several limitations of the study must be mentioned. First, we were unable to analyse long-term countrywide time trends in haematological cancer incidence because most population-based cancer registries in Spain began collecting incidence data from 1980 to 1995 and covered only 26% of the total Spanish population [34]. Nevertheless, mortality is a complex indicator that allows assessing both the effect of risk factors on cancer incidence and the impact of diagnostic and therapeutic advances on cancer survival. Second, deaths from haematological cancers were coded according to successive ICD revisions, which are not supported by the new classification of lymphoproliferative syndromes established in 1995, known as the Revised European-American Classification of Lymphoid Neoplasms [35]. Although ICD rubrics for lymphomas, multiple myeloma, and leukaemia have an overlap in several cell lines, haematological cancer death certification in Spain has been shown to be accurate from 1980 onwards [17]. However, there is no information on the quality of death certification in Spain before 1980 and we cannot rule out some degree of under-certification of haematological cancer deaths in earlier periods. To evaluate the potential impact of under-certification on the observed time trends, sensitivity analyses were performed excluding deaths during the initial quinquennia 1952-1956 and 1957-1961 and results remained virtually unchanged (data not shown).

## Conclusions

The dramatic increases in NHL and multiple myeloma mortality observed in Spain during the second half of the 20th century are best explained by a cohort effect linked to progressive improvements in the diagnosis and certification of these haematological tumours, particularly in the elderly. After full implementation of modern diagnostic procedures, the recent stabilisations or slight declines in NHL and multiple myeloma mortality rates are expected to continue in the near future. In contrast, the sustained decline in Hodgkin’s disease mortality and the levelling off in leukaemia during the last decades are mainly attributable to a period effect linked to advancements in disease treatment. Also, the consistent male excess mortality across all calendar periods and age groups points to the importance of possible sex-related genetic markers of susceptibility in haematological cancers.

## Abbreviations

APC: Annual percent change; CI: Confidence interval; ICD: International classification of diseases; NHL: Non-Hodgkin’s lymphoma.

## Competing interests

The authors declare that they have no competing interests.

## Authors’ contributions

RPB and GLA conceived and designed the study. RPB performed the statistical analysis and wrote the first draft of the manuscript. GLA contributed to the analysis and interpretation of data, and critically revised the manuscript for important intellectual content. Both authors read and approved the final manuscript.

## Pre-publication history

The pre-publication history for this paper can be accessed here:

http://www.biomedcentral.com/1471-2407/14/250/prepub

## Supplementary Material

Additional file 1: Table S1 Codes for haematological cancers from successive revisions of the International Classification of Diseases in Spain over the period 1952-2006. Click here for file

Additional file 2 **Video S1.** Rotated surface plot of age- and period-specific mortality rates of non-Hodgkin’s lymphoma by sex, Spain 1952-2006. Click here for file

Additional file 3 **Video S2.** Rotated surface plot of age- and period-specific mortality rates of leukaemia by sex, Spain 1952-2006. Click here for file
