# Supplementary material for: Changes in period and cohort effects on haematological cancer mortality in Spain, 1952-2006
Source: BMC Cancer. 2014 Apr 10;14:250. doi: 10.1186/1471-2407-14-250 (PMC4021557; doi:10.1186/1471-2407-14-250)
Supplement: Additional file 1: Table S1 — Codes for haematological cancers from successive revisions of the International Classification of Diseases in Spain over the period 1952-2006. [file 1471-2407-14-250-S1.pdf]

Supplementary Table S1 Codes for haematological cancers from successive revisions of the International Classification of Diseases in Spain over the period 1952–2006

	Revision of the International Classification of Diseases (period)			
**Haematological cancer**	**6–7th (1952–1967)**	**8th (1968–1979)**	**9th (1980–1998)**	**10th (1999–2006)**
Hodgkin's disease	201	201	201	C81
Non-Hodgkin's lymphoma	200, 202	200, 202	200, 202	C82–C85, C96
Multiple myeloma	203	203	203	C90
Leukaemia	204	204–207	204–208	C91–C95
